# Guideline concordant detection and management of depression among Alaska Native and American Indian people in primary care

**DOI:** 10.3402/ijch.v74.28315

**Published:** 2015-10-29

**Authors:** Vanessa Y. Hiratsuka, Julia J. Smith, Sara M. Norman, Spero M. Manson, Denise A. Dillard

**Affiliations:** 1 Research Department, Southcentral Foundation, Anchorage, AK, USA; 2 Centers for American Indian and Alaska Native Health, School of Public Health, University of Colorado Denver, Aurora, CO, USA

**Keywords:** depression, Alaska Native/American Indian people, primary care

## Abstract

**Background:**

A tribal health organization in Alaska implemented a primary care depression screening, detection and management initiative amongst 55,000 Alaska Native/American Indian people (AN/AIs).

**Objectives:**

(a) To describe the proportion of AN/AIs screening positive for depression with depression noted or diagnosed and proportion with guideline concordant management and (b) to assess whether management varied by patient and provider factors.

**Research design:**

Secondary analysis of electronic and paper medical record information of 400 AN/AIs.

**Measures:**

Provider variables, patient demographics and patient clinical factors were electronically queried. Manual chart audits assessed depression notation, diagnoses and management within 12 weeks of positive screening. Multilevel ordinal logistic modelling assessed management by patient and provider factors.

**Results:**

A depression diagnosis was present in 141 (35%) charts and 151 (38%) had depressive symptoms noted. Detection was higher among AN/AIs with moderate and severe depression (p<0.001). In total, 258 patients (66%) received guideline concordant management, 32 (8%) had some management, and 110 (28%) received no management. Younger patient age and increased provider tenure increased odds of management.

**Conclusions:**

Most AN/AIs screening positive for depression received initial guideline concordant management. Additional outreach to older patients and additional support for providers newer to practices appears warranted.

Arctic communities have experienced significant social and economic transitions over the past few generations that have rapidly transformed lifestyles of communities in the circumpolar north (1, 2). Indigenous circumpolar community members have socio-economic inequalities in drivers of health and wellbeing such as housing, healthcare, education and employment when compared to their non-indigenous counterparts (2, 3). Research within indigenous circumpolar communities has indicated a connection between rapid social disruption, compounded by poor access to healthcare due to rural and remoteness of indigenous communities as major factors contributing to poor mental health (1, 4–6).

An estimated 1 in 10 Americans meet the criteria for major depression (7). Depression is associated with increased use of behavioural health and medical services (8) as well as risk of suicide (9). Depression has considerable economic (10, 11) and social burden (12). The U.S. Preventative Services Task Force recommends that healthcare providers screen adults for depression and provide monitoring and follow-up services (13). Depression is persistently under-detected in primary care (14, 15). Furthermore, when depression is diagnosed and treatment is initiated in primary care, between 40 and 67% of people with major depression discontinue their antidepressant use within 3 months and fail to reach therapeutic effectiveness (16).

Management of depression is typically preceded by screening and a diagnostic assessment using standard diagnostic criteria to detect the presence of specific depressive disorders. Detection is followed by depression management decision-making to include no treatment in cases of mild adjustment disorder with depressed mood; first-, second- and third-line antidepressants; or specific psychotherapeutic approaches used alone or in combination with antidepressants (13, 17).

Members of racially and ethnically diverse populations are more likely to obtain treatment for depression in primary care than behavioural health specialty settings (18) yet, undetected and undertreated behavioural health conditions are greater among members of racial and ethnic diverse groups (17). Unfortunately, few studies have examined guideline concordant care for depression among racially and ethnically diverse primary care populations. Primary care providers and health systems are essential partners in achieving optimal clinical outcomes (13) among racially and ethnically diverse who continue to experience significant health disparities, many with respect to conditions directly affected by depression.

In the absence of effective detection or depression management, depression screening will not improve health outcomes (8). To target future improvement efforts, understanding where screening, detection and management processes may deviate from guideline recommended care is essential (19). Previous studies documented systematic variation in screening according to patient and provider factors among 47% of AN/AI adults screened according to guidelines (20). A recent cohort study of Alaska Native and American Indian (AN/AI) adults found that depression is common with rates comparable to those reported in other studies of indigenous populations (21). AN/AI adults are more likely than the general population to use the primary care system as their behavioural health system (22, 23). Hence, providing evidence-based screening, detection and management guidelines for depression is especially relevant for AN/AIs in the primary care setting.

In 2001, Southcentral Foundation (SCF) in Anchorage, Alaska implemented depression screening, detection and management guidelines to better serve the needs of the 60,000 AN/AI people in its service area. The guidelines specify annual screening of all adults seen by primary care providers followed by a diagnostic assessment to detect the presence or absence of depression, then management according to severity of depression and amenability to antidepressant medication (24).

To date, the scientific literature about depression among AN/AI people remains scarce, with less attention to guideline concordant management. We recently described factors associated with the presence or absence of annual screening as a first step in examining the effectiveness of such guidelines among AN/AI people (20). Screening varied systematically by patient and provider factors. In this manuscript, we examine the proportion of AN/AI people with positive depression screening who had depression noted or diagnosed (detected) by their primary care provider as well as the proportion with guideline concordant management 12 weeks after positive screening to determine how guideline concordance varied according to patient and provider factors. We hypothesized that guideline concordant management would be associated with gender, higher depression severity and increased service utilization among AN/AI adults, as well as increased provider tenure.

## Methods

Cross-sectional clinical encounter data from the outpatient Anchorage Native primary care clinic (ANPCC) were extracted from the electronic health records (25) of all AI/AN adults with positive screening 1 year after initiation of the depression guidelines to allow pre-screening variables to be assessed. A manual chart audit was conducted on a randomly identified sub-sample of these patients (20). The project received tribal approval and was deemed to be a quality assurance project by the Alaska Area Institutional Review Board.

### Setting

**Fig. 1:**
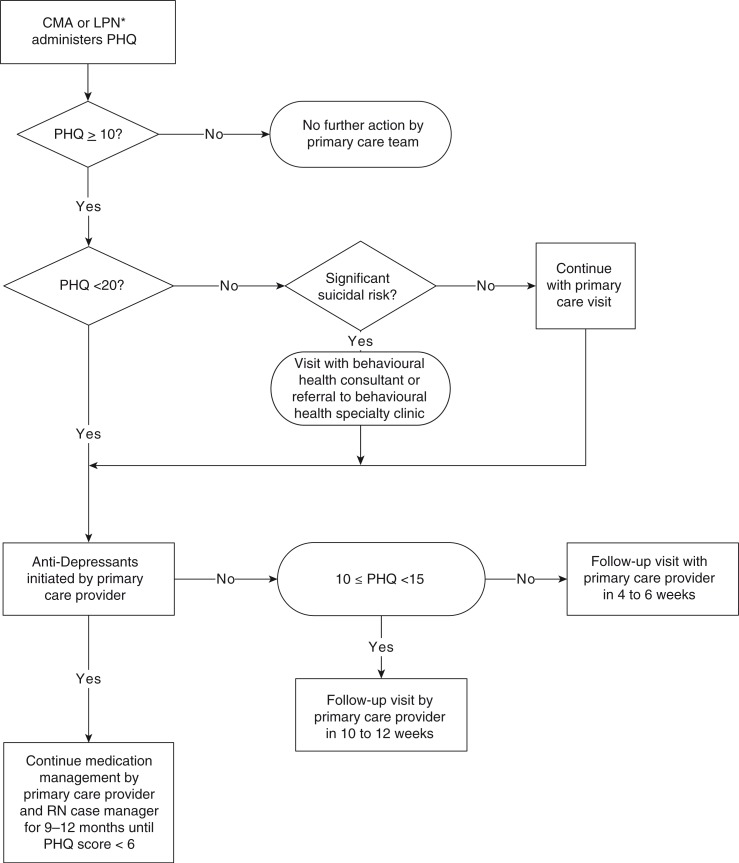
Depression screening and follow-up activities at SCF.

AN/AI people residing in Indian Health Service's Anchorage Service Unit are eligible for clinical services on a pre-paid basis (26). SCF's primary care system uses a patient-centred medical home (PCMH) model of care (27) in which each individual chooses a primary care provider and has care managed by an integrated care team (28, 29) including a co-located, master-level behavioural health consultant (BHC). The SCF depression guidelines were instituted in 2001 (24) (Fig. 1). The Patient Health Questionnaire (PHQ) is used to screen as recommended by the Institute for Healthcare Improvement (30, 31). The PHQ is derived from the Primary Care Evaluation of Mental Disorders (PRIME-MD), which has been validated in AN/AI populations (23) and has 2 components: patient questionnaire and semi-structured provider interview. The PHQ is administered in English orally and scored by Certified Medical Assistants or Licensed Professional Nurses (24) with the PHQ score documented in the paper chart along with a progress note. Select elements of the paper record (PHQ score, ICD-9 diagnostic codes, medications, provider and service codes) are then entered into the electronic health record (24). Initial management is dependent on the severity of depression according to PHQ score as mild (score 10–14), moderate (score 15–19), or severe (score 20–27) as well as the willingness of the patient to initiate antidepressant medication.

### Sample

We queried a simple random sample of 400 individuals with the following eligibility requirements: AN/AI heritage, a visit with a primary care provider between 1 March 2002 and 31 August 2003 and a PHQ score of 10 or above, age of at least 18 on the date of screening, and no diagnosis of depression nor treatment with antidepressants in the 12 months prior to positive screening. The sample size of 400 was chosen, given time and cost constraints and given *a priori* power calculations to detect 20% differences in guideline adherence for mild, moderate and severe depression with at least 80% power. The study timeframe allowed investigation of follow-up and treatment for depression immediately following a 1-year implementation period for the depression collaborative process (24) in a healthcare system that had recently transitioned to a PCMH (28).

### Procedures

Electronic medical records (EMR) were queried for provider characteristics and patient demographics as well as clinical and service factors in the year prior to screening. Paper charts were then reviewed manually to identify all visits for depression management from the date of depression screening through 12 weeks thereafter in accordance with SCF depression follow-up and treatment guidelines (Fig. 1). Chart audits assessed management information not captured electronically to include score of the PHQ item assessing suicidal ideation, symptoms of depression noted in progress note text, and patient declination of referrals or treatment options. The chart audits were performed by a psychologist and research associate using a structured form with 10% of charts audited in duplicate for quality assurance.

### Measures

Patient demographics from the EMR included age and gender. Patient clinical factors were calculated for the year prior to the first visit in the study period and included number of visits, presence of substance dependence or abuse diagnoses and the total number of the following common health conditions present: hypertension, heart disease, type II diabetes, liver disease, renal disease and pulmonary disease. Provider variables included gender and length of tenure at SCF.

The chart audit assessed the following variables for the visit when a positive depression screening was identified (referred to as the index visit): date of visit, handwriting legibility, aggregate PHQ score, PHQ item assessing suicidal ideation, assessment of plan to commit suicide, diagnosis and notation of depression in diagnostic field, diagnosis and notation of depression or depressive symptoms in text of progress note, antidepressant prescribed and referrals to BHCs or the behavioural health specialty clinic (BHSC). A depressive disorder diagnosis was operationalized as the presence of one or more of the following ICD-9 diagnoses: major depression (ICD-9: 296.0–296.9), dysthymia (300.4), adjustment disorder with depressed mood (309.00), adjustment disorder with mixed anxiety and depressed mood (309.28), or depressive disorder not otherwise specified (311.0). Depressive symptoms included depressed mood, anhedonia, difficulties concentrating, changes in appetite, insomnia or hypersomnia, fatigue, psychomotor agitation or retardation, inappropriate feelings of guilt or worthlessness, thoughts of suicide or being better off dead. Diagnosis or notation of other behavioural health disorders or psychosocial issues were assessed in the chart audit. These variables plus clinic, appointment type (in-person, phone, consultation) and appointment status (kept, not kept), were assessed at each visit to the ANPCC, BHSC and emergency room (ER) for 12 weeks after the index visit.

Initial guideline concordance was categorized into 1 of 3 levels: guideline concordant depression management, some management and no management evident. For visits beyond the index visit, diagnosis or notation of depression or another behavioural health disorder was used as an indicator of whether the visit addressed behavioural health versus a visit solely focused on non-behavioural health reasons. Manual chart audit data were entered into Microsoft Excel with 10% double-entered to check accuracy (32).

### Data analysis

Data were uploaded into SAS 9.3 (Cary, NC) for database management and descriptive analysis and exported for multilevel ordinal logistic regression analysis to M-Plus version 5.21 (Los Angeles, CA). p-Values less than 5% were considered statistically significant. We calculated agreement between coders and 10% of double-entered manual chart audits. Descriptive statistics for the index visit were calculated with differences across depression severity categories tested by the chi-square test. Descriptive statistics for patient and provider factors characteristics were calculated by the 3 levels of guideline concordance. As management may vary according to primary care provider and team, multilevel ordinal logistic modelling assessed the log-odds of management while simultaneously addressing the nesting of patients within provider. We used a multilevel model approach similar to that suggested by Hox (33). The first model fit was an unconditional model with a random intercept varying by provider and no patient or provider level factors. The intraclass correlation was calculated from the provider-level variation estimated in this model. Next, we included patient-level factors and allowed random intercepts with no provider-level factors. In the final step, we added provider-level factors in the random intercept model. Provider tenure varied by patient as it was calculated at the patient's first visit in the study period; thus, the provider tenure was included in the model at the patient level. Models were compared with the deviance test, Akaike's Information Criterion (AIC) and Bayesian Information Criterion (BIC).

## Results

Over the study period, 13% (n=945) of SCF patients screened for depression scored higher than 10 on the PHQ. In chi-square tests of association (data not shown), those in higher PHQ depression severity categories were more likely to be female (p<0.001), younger than 50 (p<0.001) and have a depression (p<0.001) or substance abuse diagnosis (p=0.003) in the 12 months prior to the patient's first visit of the study period. Further, increased odds of scoring in higher categories were associated with increased service utilization in the year prior. Of the 945 patients with positive screening, there were 865 patients with neither behavioural health diagnosis nor dispensation of a depression medication in the year prior and thus considered “newly” diagnosed with depression.

### Detection on index visit

From these 865 individuals, 400 charts were randomly selected for manual audit. Of the 400 patients, 149 (37%) scored between 10 and 14 on the PHQ indicating mild depression, 150 (38%) scored between 15 and 19 indicating moderate depression, and 101 (25%) scored 20 or higher indicating severe depression. Patients clustered within 32 different primary care providers.

On the day of the index visit with positive depression screening, a depression diagnosis was present in the diagnosis field or progress note for 141 (35%) charts. An additional 151 (38%) had no diagnosis, but had notation of depression or depressive symptoms in the progress note. Rates of depression diagnosis or notation were significantly higher among patients with moderate and severe depression (p<0.001). Of the 108 charts (27%) without diagnosis or notation of depression, 30 had illegible handwriting or were missing medical record pages where this diagnosis or notation could be present. Of the remaining 78, 14 had another behavioural health disorder or symptom noted (e.g. anxiety, substance abuse) and 19 had a psychosocial issue noted (e.g. chronic pain).

### Management according to depression severity

In Table I, the 3 levels of management are depicted according to PHQ depression severity. Among patients with mild and moderate PHQ depression severity, roughly two-thirds had concordant management. Of those, 65 and 75% received antidepressants (mild and moderate, respectively) and the remainder attended at least one follow-up visit in accordance with Fig. 1. Among patients scoring in the most severe PHQ category, treatment guidelines suggest an assessment for suicidal ideation with immediate referral to BHSC or a BHC, and then the prescription of an antidepressant medication or behavioural health visit within 6 weeks is specified for additional management. Among the 101 patients with severe depression symptoms, 73 (72%) were assessed for suicide or referred to BHSC or a BHC. A total of 62 (61%) of patients with severe depression symptoms received guideline concordant management with 55 (89%) receiving antidepressants. Other patients (n=24, 24%) with severe depression symptoms received follow-up, though not concordant with guidelines.

**Table I:** Level of guideline concordant management by depression severity^a^

	Mild depression (n = 149)		Moderate depression (n = 150)		Severe depression (n = 101)	
			
	n	%	n	%	n	%
No management	50	34	45	30	15	15
PCC visit without behavioural health addressed	26	17	21	14	5	5
No PCC^b^ or BHSC^c^ visit	24	34	24	16	9	9
Declined antidepressant prescription	2	1	9	6	0	0
Missed scheduled appointment	2	1	5	3	1	1
Emergency room visit	4	3	3	2	4	4
Some management^d^	0	0	8	5	24	24
PCC visit outside of specified time frame	0	0	6	4	21	21
BHSC visit outside of specified time frame	0	0	2	1	3	3
Guideline concordant management	99	66	97	65	62	61
Antidepressant prescribed	64	47	73	53	55	54
Visit within specified time frame^e^	35	24	24	16	7	7
PCC	26	18	18	12	2	2
BHSC	9	6	6	4	5	5

a According to total Patient Health Questionnaire score (mild = score 10–14; moderate = score 15–19; severe = score 20 or higher);

b primary care clinic;

c behavioural health specialty clinic;

d behavioural health addressed at visit outside of time frame specified in Fig. 1;

e behavioural health addressed at visit within time frame specified in Fig. 1.

In total, 258 patients (66%) were managed concordant with guidelines, 32 (8%) had some management and 110 (28%) received no management. Among these latter patients, 52 visited the ANPCC, but there was no documented follow-up for behavioural health issues. Eleven patients refused medication on the screening day; 8 had appointments that were not kept; 7 had visits only in the ANMC ER; 40 had no scheduled appointments in the 12 weeks following screening.

### Factors associated with concordant management, some management, or no management

In Table II, rates of initial concordant management are stratified by patient and provider factors. Table III presents the results of the multilevel ordinal logistic regression. Younger age (18–34 years) was associated with increased likelihood of management as compared to older age (35–49 years, 50–97 years borderline significant). Patient gender, number of physical conditions and number of visits in the year prior to the index bore no relationship to management. The inclusion of substance abuse did not improve statistical fit, likely due to small cell sizes, though when included it was not significant (p=0.757). Additionally, increased provider tenure was associated with increased management. For instance, among patients of providers with 5 or more years of experience in the clinic, 73% received guideline concordant care as compared to 55% of patients of providers with less than 2 years of tenure. There was no significant residual variation across providers or effect for provider gender.

**Table II:** Guideline concordant management by patient and provider factors

	No management (n=110)		Some management (n=32)		Guideline concordant management (n=258)	
			
	n	%	n	%	n	%
Patient factors^a^						
PHQ depression severity^b^						
Mild (score 10–14)	50	34	0	0	99	66
Moderate (score 15–19)	45	30	8	5	97	65
Severe (score 20 or higher)	15	15	24	24	62	61
Gender						
Female	86	29	21	7	194	65
Male	24	24	11	11	64	65
Age^b^						
18–34 years	39	24	5	3	118	73
35–49 years	49	31	18	12	89	57
50–97 years	22	27	9	11	51	62
Number of physical conditions^c^						
None	77	28	20	7	182	65
1 or more	33	27	12	10	76	63
Number of visits in the year prior to index visit						
None	30	28	9	8	70	64
1–7	47	25	12	6	133	69
8 or more	33	33	11	11	55	56
Provider factors^a^						
Provider gender						
Female	74	31	15	6	149	63
Male	36	22	17	11	109	67
Provider tenure						
Less than 2 years	22	36	6	10	34	55
2–5 years	66	30	17	8	140	63
5 or more years	22	19	9	8	84	73

Note: Substance abuse not shown due to small cell sizes.

a At patient's first visit with positive depression screening during the study period of March 2002 and August 2003;

b significant association (p<0.05) according to the chi-square test of association;

c hypertension, heart disease, diabetes, liver disease, renal disease and pulmonary disease.

**Table III:** Random intercept ordinal logistic regression on management (no management, some management, guideline concordant management)

	Estimate	Standard error	p-Value	Odds ratio	OR 95% CI
Patient factors^a^					
PHQ screening score	0.039	0.024	0.098	1.04	0.99–1.09
Gender (reference female)	0.072	0.161	0.654	1.08	0.78–1.48
**Age (reference 18–34 years), years**					
** 35–49**	**−0.678**	**0.266**	**0.011**	**0.51**	**0.30–0.86**
** 50–97**	**−0.253**	**0.132**	**0.055**	**0.78**	**0.60–1.01**
Number of physical conditions^b^	0.003	0.181	0.985	1.00	0.70–1.43
Number of visits in the year prior to first visit in study period	−0.024	0.016	0.133	0.98	0.95–1.01
Provider factors^a^					
Provider gender (reference female)	0.355	0.200	0.076	1.43	0.96–2.11
**Provider tenure** c	**0.095**	**0.042**	**0.025**	**1.10**	**1.01–1.20**
Provider-level variation					
Random intercept variation	<0.001	<0.001	0.548	n/a	n/a

a At patient's first visit during the study period of March 2002 and August 2003;

b hypertension, heart disease, diabetes, liver disease, renal disease and pulmonary disease;

c the same provider would have different tenure for different patients, varying according to the patient's first visit date. Thus, the variable was entered in the model at the patient level.

## Discussion

Our study revealed that 73% of AN/AI adults screening positive had depression noted in their medical records. Another 8% had another behavioural health disorder or symptom noted to include the possibility of a pain disorder. Guideline concordant management then occurred 66% of the time with 8% receiving some management and an additional 5% offered management but missed appointments or declined antidepressant medication. Unlike screening, variation in management was affected by relatively few patient and provider factors, namely patient age and provider tenure.

The majority of AN/AI adults who screened positive received guideline concordant management in the 12 weeks after positive screening. These findings demonstrate the ability of health systems to successfully implement depression detection and management initiatives for AN/AI adults in primary care (34), within a PCMH model (28, 29). Non-guideline concordant management included absence of direct follow-up, missed appointments and the failure to address behavioural health issues during subsequent health encounters. Older patients were less likely to receive guideline concordant management. Newly hired providers were less likely to follow guidelines, a finding consistent with previously identified barriers to depression management, including provider lack of familiarity with behavioural health treatment and a tendency to treat other conditions primarily per provider comfort. Newer providers may also fail to document provided education when customer–owner declines formal treatment (35). It should be noted that within the ANPCC, patient centred care is a core tenet of practice. Thus, a patient may decline care or define a treatment plan outside of the clinical guideline. Starks et al. found that following depression screening, AN/AI adults expressed interest in discussing the diagnosis with their family and wanted information about various clinical and non-clinical treatment options prior to commencing treatment (36). Patient preference for non-clinical treatment (e.g. pastoral counselling, traditional healing) could account for discrepancies in guideline concordant management such as a delay in treatment or refusal of clinical treatment either by not attending treatment appointments or not scheduling follow-up care.

Several limitations of this study warrant acknowledgement. The sample was restricted to individuals receiving primary care from 2002 to 2003 at the ANPCC and results may not generalize to other regions or practices. Depression screening and coordination for follow-up care occurred in English, the language predominantly used at the ANPCC. Preference for care using a non-English language may have impacted receipt of guideline concordant care, particularly among older patients who may be more likely to use an AN/AI language. Provider handwriting was illegible for some fields in 32 (9%) charts; an additional 35 charts (9%) had relevant pages of the paper record missing for the index visit. The study did not assess effectiveness of treatment with respect to reduction in symptoms, dosage of antidepressant administered, or management beyond the 12-week period. Information about important demographic and psychosocial variables (e.g. rural/urban; education level attainment; household income; employment status; social support) related to depression treatment in other populations were not consistently recorded in the paper medical record or were overwritten electronically and thus not assessed. We were unable to determine the degree to which non-concordant care was due to provider or patient choice beyond any reasons documented in the record. Other AN/AI healthcare systems typically have very few behavioural health resources (35) and most do not offer same-day access to care.

This study has several unique strengths. Studies of depression treatment in non-research settings are scarce, as are behavioural health studies of AN/AI patients seen in urban settings. Our sample size was large, affording robust analysis of associated factors. The availability of same-day primary care and behavioural health care allowed us to examine management practices without constraint of service availability. Finally, we assessed the performance of a primary care health system using clinical records rather than patient self-report measures.

Our findings indicate that improving the rate of guideline concordance promises to significantly impact patient well-being. Additional outreach to older patients appears warranted, as does additional support for providers newer to primary care practices. Future research among AN/AI people should not only examine the effectiveness of management in resolving depression and subsequent impacts on other health domains but also address how patient and provider decision-making (35) affects outcomes. Additionally, patient and provider communication should be considered, particularly the influence on language used in the health encounter and language preferred by the patient. The effects of socio-economic inequalities and subsequent changes in AN/AI traditional lifestyles as factors negatively impacting mental health and culturally resiliency factors should also be considered in future research on depression treatment and management. Finally, research in other AN/AI settings operating under different healthcare models will better distinguish among the differential effects of the availability, implementation and outcomes of depression management (37, 38).
